# Circatidal gene expression in the mangrove cricket *Apteronemobius asahinai*

**DOI:** 10.1038/s41598-019-40197-2

**Published:** 2019-03-06

**Authors:** Aya Satoh, Yohey Terai

**Affiliations:** SOKENDAI (The Graduate University for Advanced Studies), Department of Evolutionary Studies of Biosystems, Shonan Village, Hayama, Kanagawa 240-0193 Japan

## Abstract

The mangrove cricket *Apteronemobius asahinai* is endemic to mangrove forest floors. It shows circatidal rhythmicity, with a 12.6-h period of locomotor activity under constant conditions. Its free-running activity also has a circadian component; *i.e*. it is more active during the subjective night than during the day. In this study, we investigated rhythmic gene expression under constant darkness by RNA sequencing to identify genes controlled by the biological clock. Samples collected every 3 h for 48 h were analysed (one cricket per time-point). We identified 284 significant circatidal cycling transcripts (period length 12–15 h). Almost half of them were annotated with known genes in the NCBI nr database, including enzymes related to metabolic processes and molecular chaperones. There were less transcripts with circadian rhythmicity than with circatidal rhythmicity, and the expression of core circadian clock genes did not show significant rhythmicity. This may reflect the nature of the mangrove cricket or may be due to the paucity of the sampling repeats: only two periods for circadian cycle with no replications. We evaluated for the first time the rhythmic transcriptome of an insect that shows circatidal rhythmic activity; our findings will contribute to future studies of circatidal clock genes.

## Introduction

Diverse biological phenomena, such as eclosion^1^, stridulation activity^2^ and flight activity^3^, occur at specific times of day. These rhythmicities are controlled by the circadian clock, a biological clock with a period of approximately 24 h. The molecular mechanisms of the circadian clock are known; transcriptional–translational feedback loops involving several clock genes underlie the oscillation of the circadian rhythm^4^. The circadian clock controls the rhythmic expression of a large number of genes (clock-controlled genes; CCGs), which drive many rhythmic phenomena. For example, in the mouse liver, ~9% of all transcripts show robust circadian cycling^5^. Circadian and diurnal transcriptional rhythms have been demonstrated using microarray analyses and, more recently, RNA sequencing (RNA-Seq), in several species, including circadian clock model species, such as *Drosophila*^6^ and mouse^7^, and non-model species, such as sea anemone^8^ and mosquito^9^.

In addition to or instead of a circadian rhythm, some intertidal animals show an endogenous activity rhythm with a period of approximately 12.4 h, which is synchronised to the cycle of tidal flooding and ebbing; *i.e*. a circatidal rhythm^10,11^. Researchers have long discussed whether the circatidal rhythm is underlain by the same mechanism as the circadian clock or by some other mechanism; *i.e*. a circatidal clock^12,13^. Circatidal rhythmic activity can be induced by a hypo-osmic shock in the shore crab *Carcinus maenus*, with no concomitant change in the phase of the circadian rhythm; thus, separate mechanisms appear to control circadian and circatidal rhythmicity in this crab^14^. In contrast, several phenomena contributing to the free-running activity rhythm of the fiddler crab *Uca* cannot be explained by the circatidal clock^15^. These phenomena include the desynchronisation of two alternate circatidal peaks (associated with day and night high tides, respectively) or temporal or permanent disappearance of one or the other peak. Based on these findings, the following hypothesis has been proposed; the circatidal rhythm is controlled by two separate oscillators with a period of 24.8 h; *i.e*. oscillators possessing fundamentally the same period as the circadian clock, which are loosely coupled at 180° out of phase^15^. The putative molecular mechanism for these two hypotheses may be different. While the molecular mechanism should be fundamentally the same with the circadian clock under the assumption of oscillators with a period of 24.8 h^15^, the circatidal clock assumes some kind of molecular loop, such as the transcriptional–translational feedback loops, rotating with the period of 12.4 h. The putative mechanism of the circatidal clock does not necessarily exclude the possibility that circadian clock genes involve into this mechanism, but if so they should function differently with the case of the circadian clock. Although recent advances in molecular techniques have enabled new approaches to investigate these matters, little is known about the molecular mechanisms underlying the circatidal clock^16,17^.

Some insects depend on intertidal regions for foraging and/or reproduction^18^, but the circatidal rhythms of insect activity have not been examined thoroughly^19–21^. An exception is that of the mangrove cricket *Apteronemobius asahinai*, which is endemic to mangrove forest floors. Mangrove forests are influenced by tidal flooding and ebbing, and the activity rhythm of the mangrove cricket is defined by the tidal cycle. Under constant conditions, it shows a clear circatidal rhythm in its locomotor activity, with a period of approximately 12.6 h^22,23^. The cricket is active during expected low tides and inactive during expected high tides. In addition to this clear circatidal rhythm, its locomotor activity shows a circadian rhythm; *i.e*. it is more active during expected night low tides than during day low tides^22^. Recent studies have demonstrated that the circadian, but not the circatidal, component of locomotor activity under constant darkness (DD) is disrupted by silencing of the expression of circadian clock genes, *period* or *Clock*, by RNAi^24,25^. This finding indicates that the molecular components of the circatidal clock differ from those of the circadian clock in the mangrove cricket. The identification of circatidal clock genes in this insect is thus required.

Few studies have investigated rhythmic expression in intertidal organisms. In the case of the intertidal mussel *Mytilus californianus*, ~85% of the rhythmic transcripts follow a diel expression pattern, whereas only <10% of these transcripts follow a tidal pattern^26^. In contrast, the intertidal limpet *Cellana rota* exhibits more transcripts with tidal expression patterns than with diel expression patterns in its transcriptome^27^. Because these previous whole-genome expression studies of intertidal organisms involved the analysis of individuals sampled from simulated and natural tidal environments, the results included not only CCGs, but also genes whose expression oscillated in response to environmental changes. Sample collection for evaluation of the rhythmic expression of clock genes and CCGs must be performed under constant conditions.

In this study, we investigated the rhythmic expression of genes in the mangrove cricket under DD by RNA-Seq. Any rhythmic expression identified under constant conditions is expected to be underlain by the biological clock. The present study provides the first rhythmic expression pattern on an insect that shows circatidal rhythmic activity and will facilitate further investigation of circatidal clock genes.

## Results

### Transcriptome analyses

To perform *de novo* transcriptome assembly, we analysed two adult female crickets collected from a mangrove forest at high tide and low tide. In total, 54,725 transcripts with a maximum length of 14,723 bp, a minimum length of 300 bp, and an average length of 962 bp was obtained by *de novo* assembly. The transcriptome N50 was calculated to be 1,472 bp. Among the 1,066 arthropodal universal orthologues, 956 genes (89.68%) were identified completely and 87 genes (8.16%) were identified partially in our transcriptome.

**Figure 1:**
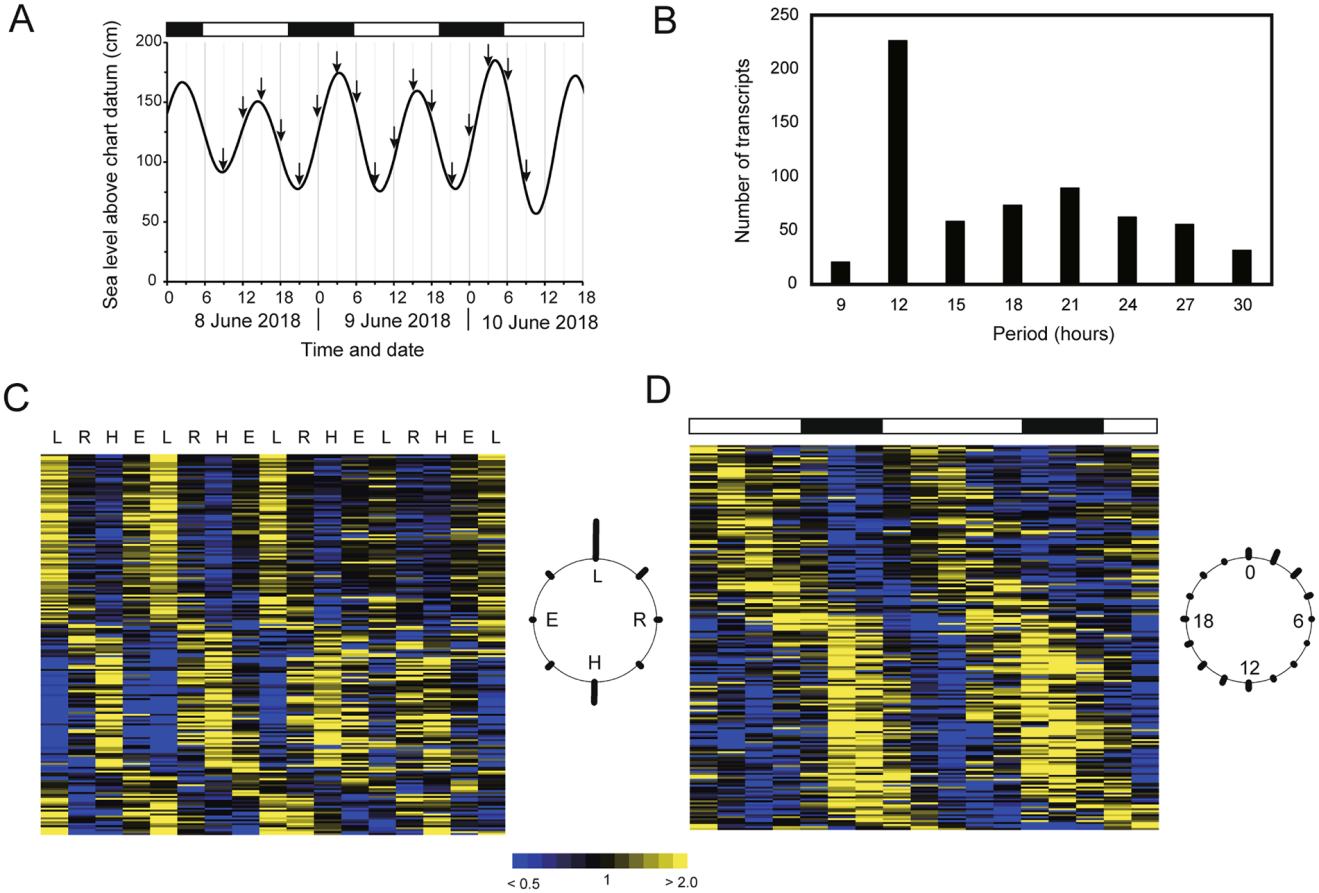
Rhythmic expression of genes in the mangrove cricket under constant darkness. (**A**) Sampling schedule. Arrows indicate sea level in the field at each sampling time point. Samples were collected under constant darkness at a constant temperature (25 °C). (**B**) Frequency of period lengths (in hours) among 614 significantly cycling transcripts. (**C**,**D**) Heat maps of the expression of 284 circatidal (period length, 12–15 h) transcripts (**C**) and 206 circadian (21–27 h) transcripts (**D**). Colour scale from blue to yellow represents the fold increase in expression compared with the median. Data are sorted by phase values, *i.e*. times of peak expression, calculated by JTK_Cycle. Circular plots at right show the frequency distribution of the times of peak expression in the circatidal (**C**) and circadian (**D**) time frames. White and black bars indicate expected day and night, respectively (**A**,**D**). L, expected low tide; R, expected rising tide; H, expected high tide; E, expected ebb tide (**C**).

To identify biological clock-regulated transcripts, we performed transcriptional analyses using the above *de novo* assembly as a reference with samples collected every 3 h for 48 h under DD (Fig. 1A). This time frame covered four tidal and two daily periods. We detected 614 cycling transcripts (*p* < 0.05) among 23,057 transcripts (i.e., transcripts ≥500 bp long with the first quartile of 17 expression values >0) by looking for transcripts with a period length of 9–30 h (2.7%; Fig. 1B). Among them, transcripts with a cycling period of 12 h were the most frequent (Fig. 1B). We identified 284 significant circatidal cycling transcripts (period length 12–15 h, *p* < 0.05; Fig. 1C) and 206 significant circadian cycling transcripts (21–27 h, *p* < 0.05; Fig. 1D). The distribution of the peak phases of circatidal cycling transcripts deviated significantly from uniformity (*p* < 0.01), and the expression of most transcripts peaked at low or high tide (Fig. 1C). The distribution of the peak phases of circadian-cycling transcripts also deviated significantly from uniformity (*p* < 0.01), and slightly more transcripts peaked from midnight to dawn or from noon to afternoon (Fig. 1D).

### Functional annotation of cycling transcripts

**Figure 2:**
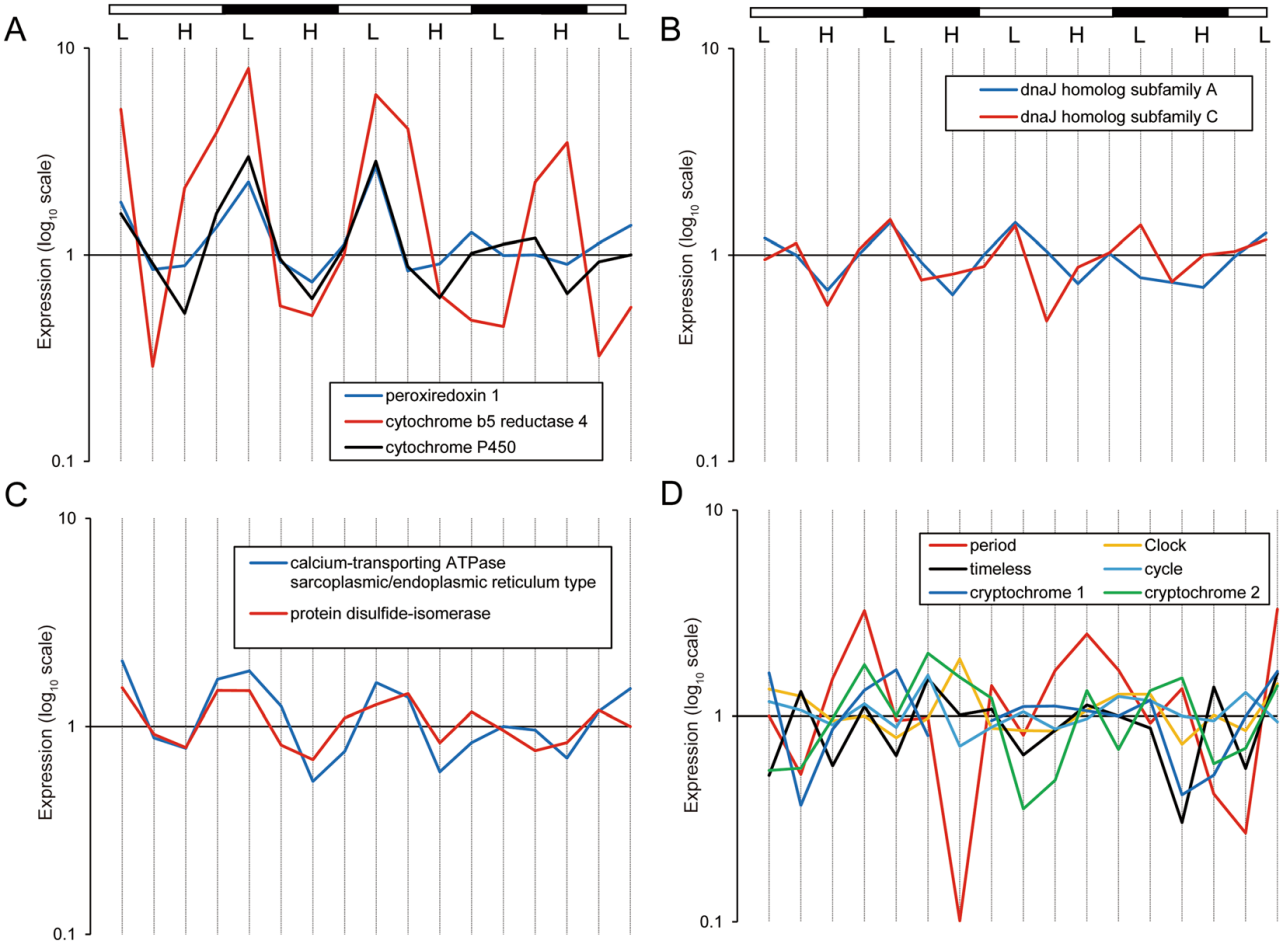
Expression profiles of selected genes in the mangrove cricket. Expression profiles were shown for genes related to metabolic prosses (**A**), molecular chaperones (**B**), genes related to the endoplasmic reticulum (**C**), and core circadian clock genes (**D**). Expression values were normalised to the median expression of the corresponding genes. White and black bars indicate expected day and night, respectively. L, expected low tide; H, expected high tide.

Among 284 circatidal cycling transcripts, 158 (56%) were annotated with known genes in the NCBI nr database (Supplementary Table S1). We identified genes encoding enzymes related to metabolic processes, such as *peroxiredoxin 1*, *cytochrome b5 reductase 4*, and *cytochrome P450* (Fig. 2A), and molecular chaperones *dnaJ homologue subfamily A member 2-like* and *dnaJ homologue subfamily C member 6* (Fig. 2B), the expression of which peaked at subjective low tide. We also identified genes encoding proteins related to the endoplasmic reticulum, such as *calcium-transporting ATPase sarcoplasmic/endoplasmic reticulum type* and *protein disulphide-isomerase* (Fig. 2C), and proteins that may be related to regulation of gene expression, such as zinc-finger proteins. Among 206 circadian cycling transcripts, 116 (56%) were annotated with known genes in the NCBI nr database (Supplementary Table S2). The annotation lists for circatidal and circadian transcripts did not contain the core circadian clock genes. The orthologues of core clock genes, with the exception of *period*, showed arrhythmic expression profiles. The core clock gene *period* exhibited a diurnal expression pattern with a 21-h period, peaking late in the subjective day (Fig. 2D), but no significance was detected (*p* = 0.54).

## Discussion

The present study revealed the first rhythmic transcriptome of an insect with a circatidal activity rhythm: we identified a few hundreds of significant circatidal cycling transcripts (period length 12–15 h), which are expected to be underlain by the biological clock. We obtained 54,725 transcripts by the *de novo* transcriptome assembly of the mangrove cricket. Considering that the number of genes in *Drosophila* have been estimated to be almost 14,000^28^, our transcripts appear to contain non-coding RNA and fragmented transcripts of genes. Indeed, only half of circatidal cycling transcripts could be annotated with known genes in the NCBI nr database. This fact hints at the possibility that some non-coding RNA oscillate with the circatidal period, as suggested by transcriptomic analysis of *Drosophila* brain, in which at least ~5% of the circadian cycling genes were non-coding RNA^6^.

The expression of genes encoding enzymes related to metabolic processes, such as *peroxiredoxin 1*, showed circatidal rhythmicity. Peroxiredoxins possess peroxidase activity; *i.e*. they reduce reactive oxygen species (ROS), such as hydrogen peroxide, which cause oxidative damage to biomolecules^29^. While peroxiredoxin reduces ROS, it is oxidised. Intriguingly, the overoxidation cycle of peroxiredoxin in the marine crustacean *Eurydice pulchra* exhibits a clear circatidal pattern under free-running conditions^30^. Although the rhythmicity of peroxiredoxin overoxidation in the mangrove cricket is unknown, the expression of *peroxiredoxin 1* showed a circatidal rhythm, peaking at subjective low tide; *i.e*. the period of activity. If the oxygen consumption is higher during the period of activity than during the rest period, then the risk of oxidative damage may be higher during the period of activity, which may explain the high expression of *peroxiredoxin 1* at subjective low tide. *Cytochrome b5 reductase*, which has an antioxidant effect on the plasma membrane^31^, and c*ytochrome P450*, which is involved in the metabolism of drugs, carcinogens, and steroids in humans^32^, also showed a circatidal expression pattern in the mangrove cricket. The importance of metabolic processes for coping with the tidal cycle was also supported by metabolomic analysis of an intertidal mussel, which revealed a strong relationship between oscillations in metabolite abundance and the environmental cycle of emersion and immersion in intertidal ranges^33,34^.

Twelve-hour cycling transcripts have been also found in the transcriptomes of mouse and nematodes, which do not inhabit intertidal regions^35,36^. The 12-h cycling genes in the mouse liver were highly enriched for endoplasmic reticulum (ER)- and metabolism-related biological pathways. We also found that ER-related genes, such as *calcium-transporting ATPase sarcoplasmic/endoplasmic reticulum type* and *protein disulphide-isomerase*, and molecular chaperones, as well as metabolism-related genes, exhibit a 12-h expression cycle in the mangrove cricket. Intriguingly, the mammalian 12-h rhythm is cell-autonomous, driven by a dedicated 12-h pacemaker distinct from the circadian clock, and the distinctive 12-h clock have been hypothesized to be conserved between mouse and nematodes, presumably having its origin in the circatidal clock of the common ancestor in ancient oceans^36^. If the 12-h clock is conserved between nematodes and mammals, it may also have been in insects. The inland cricket is clearly the ancestor of the mangrove cricket and the mangrove cricket has returned to the intertidal environment. Thus, the circatidal clock genes, which were long dormant in the insect genome, may have been awoken by the need to adapt to the intertidal zone.

Although the molecular bases of the circatidal clock of the mangrove cricket appear to differ from those of the circadian clock, transcription factors must be involved in regulation of the expression of downstream circatidal cycling genes, such as the circadian transcription factor *BMAL1*^37^. In the present study, we identified genes encoding transcription factors and zinc-finger proteins, which function as DNA-binding proteins^38^. These homologues might encode transcription factors that regulate the expression of circatidal clock genes, driving the molecular loop with the period of 12.4 h. Further studies, for example using ChiP-Seq, are needed to reveal the function of these candidate genes, which will give an answer to the question whether the different mechanism from the circadian clock involve into the expression of the circatidal rhythm or not.

More transcripts cycled with a circatidal than with a circadian rhythmicity, and we could not find significant rhythmic expression of core circadian clock genes in the present study. These findings may suggest that the biogenic activity of the mangrove cricket is influenced strongly by environmental changes related to the tidal cycle. The lack of a significant diel or tidal gene expression pattern in core circadian clock genes has been also reported in an intertidal limpet^27^ and in the horseshoe crab^39^. Alternatively, we cannot deny the possibility that the present results are due to the paucity of the sampling repeats: only two periods for circadian cycle with no replications. In the mangrove cricket, the previous study have revealed by using quantitative real-time PCR that the relative abundance of *period* mRNA under a light:dark (LD) cycle is higher at the beginning of the dark phase than in the light phase^24^. Our results are basically consistent with this finding, although the time of peak expression shifted to the late afternoon probably because of the <24-h free-running period. Nevertheless, we could not detect the significant rhythmicity. Additional analyses with more sampling time points and replicates will clarify the expression pattern in core circadian clock gene and their CCGs in the mangrove cricket.

In this study, we identified 284 genes that show circatidal oscillation under constant conditions. Although several previous studies have involved the investigation of genes with tidal oscillation in expression in intertidal organisms, the core genes of the circatidal clock remain elusive. Our results will facilitate further investigation of circatidal clock genes in insects and provide genetic resources for exploration of the genetic mechanism of the circatidal clock.

## Methods

### Cricket sampling

To conduct *de novo* transcriptome assembly, we collected two adult female crickets from a mangrove forest in Ginoza, Okinawa Prefecture, Japan; one was collected at high tide (7:30 AM) on 27 May 2017 and the other was collected at low tide (1:30 PM) on the same day. The crickets were immediately preserved in RNAlater (Waltham, MA, USA).

More than 40 adult female crickets were collected from the same mangrove forest in Ginoza on the morning of 7 June 2018 to analyse transcript expression rhythmicity. They were housed in a plastic case with wet cotton on the bottom and dried food (Oriental Yeast, Tokyo, Japan) in plastic Petri dishes. Crickets were placed under DD at a constant temperature (25 °C) until dusk on the day of collection. Starting at 9:00 AM the next morning, a cricket was collected every 3 h for 48 h under dim red light (17 time points; Fig. 1A). The samples were immediately frozen in liquid nitrogen and stored at −80 °C. The first sampling time point almost corresponded to low tide, the second to the rising tide, the third to high tide, and the fourth to ebb tide, and these correspondences between time points and tides were maintained until the last time point (Fig. 1A).

### RNA extraction, cDNA library construction and sequencing

Total RNA was extracted from the heads of the crickets using an RNeasy Mini Kit (QIAGEN, Hilden, Germany). The NEBNext Poly(A) mRNA Magnetic Isolation Module and NEBNext Ultra Directional RNA Library Prep Kit for Illumina (New England Bio Lab, Ipswich, MA, USA) were used to construct cDNA libraries. Paired-end (125-bp) sequencing (RNA-Seq) was performed on the Illumina HiSeq2500 platform.

### Assembly, rhythmicity analysis, and annotation

After removal of adaptor sequences and low-quality reads, the RNA-Seq reads (10 Gb) obtained from a cricket collected at high tide in 2017 were assembled *de novo* using the CLC genomic workbench (https://www.qiagenbioinformatics.com/). To cover the entire range of the expressed genes, including those expressed only at low tide, the RNA-Seq reads (5 Gb) from a cricket collected at low tide were mapped to the assembled transcripts of a high-tide individual; the unmapped reads were also assembled *de novo*. The transcripts obtained by the above two *de novo* assemblies were combined and used as a reference. The completeness of the transcriptome was assessed using gVolante (https://gvolante.riken.jp/index.html), an online interface for assessment of the completeness of sequence datasets by means of the pipeline BUSCO_v2^40^.

RNA-Seq reads from 17 crickets (*i.e*. 17 time points under DD: 5.2–7.1 Gb) were mapped to the reference transcripts, and the number of reads per kilobase per million mapped reads (RPKM) were calculated for each transcript. The RPKM values were subjected to quantile normalisation^41^. Rhythmicity was analysed using the JTK_Cycle algorithm, a non-parametric algorithm developed to identify rhythmic components in large, genome-scale datasets and to estimate their period lengths, phases, and amplitudes^42^. Transcripts ≥500 bp long with the first quartile of 17 expression values >0 were used for the rhythmicity analysis. We searched for transcripts with period lengths of 9–30 h and used permutation-based *p* values (ADJ.P), which were calculated by the JTK_Cycle, to select significantly cycling transcripts. Transcripts whose expression showed significant rhythmicity with periods of 12–15 h (*p* < 0.05) were identified as circatidal cycling transcripts, and those with periods of 21–27 h (*p* < 0.05) were identified as circadian cycling transcripts. The transcripts were annotated using BLASTX search^43^ with default settings against the NCBI non-redundant protein sequence (nr) database using Blast2GO software^44^. To identify homologues of the core circadian clock genes, the reference transcripts were blasted against the sequences of *period* (accession no. AB550300), *Clock* (AB894249), *timeless* (AB548625), *cycle* (AB762416), *cryptochrome 1* (LC202047), and *cryptochrome 2* (LC202048). Because the phases, i.e., the time at which expression was highest in the circatidal or circadian time frame, are circular, Kuiper’s test of uniformity in the R package ‘circular’^45^ was implemented to verify the uniformity of the distribution of phase values.

## Supplementary information

Supplementary Table S1

Supplementary Table S2

## Data Availability

All raw data were deposited in the DDBJ Sequenced Read Archive under accession numbers DRX151508–DRX151526.
